# Application of quantum computing to a linear non-Gaussian acyclic model for novel medical knowledge discovery

**DOI:** 10.1371/journal.pone.0283933

**Published:** 2023-04-05

**Authors:** Hideaki Kawaguchi

**Affiliations:** Quantum Computing Center, Keio University, Yokohama, Japan; Jeonbuk National University, REPUBLIC OF KOREA

## Abstract

Recently, the utilization of real-world medical data collected from clinical sites has been attracting attention. Especially as the number of variables in real-world medical data increases, causal discovery becomes more and more effective. On the other hand, it is necessary to develop new causal discovery algorithms suitable for small data sets for situations where sample sizes are insufficient to detect reasonable causal relationships, such as rare diseases and emerging infectious diseases. This study aims to develop a new causal discovery algorithm suitable for a small number of real-world medical data using quantum computing, one of the emerging information technologies attracting attention for its application in machine learning. In this study, a new algorithm that applies the quantum kernel to a linear non-Gaussian acyclic model, one of the causal discovery algorithms, is developed. Experiments on several artificial data sets showed that the new algorithm proposed in this study was more accurate than existing methods with the Gaussian kernel under various conditions in the low-data regime. When the new algorithm was applied to real-world medical data, a case was confirmed in which the causal structure could be correctly estimated even when the amount of data was small, which was not possible with existing methods. Furthermore, the possibility of implementing the new algorithm on real quantum hardware was discussed. This study suggests that the new proposed algorithm using quantum computing might be a good choice among the causal discovery algorithms in the low-data regime for novel medical knowledge discovery.

## Introduction

### Background

The utilization of medical data, which is increasing with the digitalization of medicine, has been attracting attention [1]. As a method of utilizing medical data so far, clinical trials such as randomized controlled trials have been conducted to establish scientific evidence. However, it has been reported that there are problems associated with clinical trials, including the strict selection/exclusion criteria, as well as time, cost, and ethical restrictions required for their implementation [2]. By contrast, real-world medical data, which are secondary data collected from clinical environments, have been attracting attention as data that are rapidly increasing with the digitalization of medicine, for example, disease registries, electronic medical record data, and claim data containing details of medical procedures [2, 3]. Real-world medical data can be collected from a wide range of patients with lower cost, time, and ethical constraints than those of clinical trials.

Currently, the main method for analyzing real-world medical data is “causal inference,” in which the direction of a certain causal relationship is determined and then the causal effect, which represents the strength of the causal relationship, is estimated. As long as the direction of the causal relationship is known in advance, the methods for estimating causal effects have continued to evolve significantly in recent years with the improvement in statistical methods [4–8]. However, in the medical field where there are many uncertain cases, it is difficult to determine the direction of causality in advance in many situations. In these situations, “causal discovery,” which detects causal relationships from data, is important for the discovery of new knowledge [9].

The causal discovery algorithm is a method for identifying causal graphs that represent causal relationships among variables by determining the direction of causal relationships according to the data, with some assumptions [9]. A wide range of applications for causal discovery algorithms has been anticipated in biology and medicine [10], including use in neuroscience [11] and epidemiology [12].

Particularly in the case of real-world medical data, the number of variables to be handled is so large that it is not realistic to exhaustively confirm causal relationships in advance, and thus, the importance of causal discovery methods is expected to grow stronger. On the other hand, situations in which a reasonable causal relationship cannot be detected due to insufficient sample size, for example, when targeting rare diseases or emerging infectious diseases, are anticipated [13]. For a wider range of medical applications, it is important to develop new causal discovery algorithms that are highly accurate even for small numbers of data.

### Quantum computing

Recently, quantum computing has been attracting attention as a new information technology, including its application to machine learning, known as quantum machine learning [14, 15]. For example, some quantum machine learning algorithms have been proposed that outperform their conventional counterparts for certain classes of problems [16]. In conventional (or classical) computers, the state of a bit is either 0 or 1; however, quantum computers use quantum bits, or qubits, which can take superpositions of 0 and 1, and quantum mechanical principles such as quantum entanglement for information processing. Because qubits are sensitive to noise and the superposition state is broken after a certain period of time, the fault-tolerant quantum computer that can correct errors caused by noise is required; however, there are still many technical and essential problems to be solved for realizing the fault-tolerant quantum computer. By contrast, a quantum computer called noisy intermediate-scale quantum (NISQ) computer, which does not have an error correction function, has been realized in the last few years [17, 18].

A well-known example of quantum machine learning that can be implemented with a NISQ computer is the quantum kernel, which applies quantum computing to kernel methods [15, 19, 20]. For an experiment on quantum kernels using a NISQ computer, the work of applying quantum kernels to support vector machine frameworks is known [15]. Kernel methods embed data in a high-dimensional feature space to facilitate analysis, whereas quantum kernels use quantum circuits to construct the kernel. With the use of the superposition state and the information in the high-dimensional feature space, it is expected that quantum computers might efficiently construct kernels that are difficult to represent with conventional computers. For example, it has been demonstrated that applying quantum kernels to support vector machines can successfully classify a specific data set that cannot be efficiently classified by conventional models [16].

### Related work

Traditional causal discovery algorithms involve constraint-based methods such as Peter-Clark [21], and score-based methods such as Greedy Equivalence Search [22]. These methods have the limitation of being unable to uniquely identify the structure of the causal graph [21, 23]. On the other hand, the linear non-Gaussian acyclic model (LiNGAM) uniquely identifies the structure of causal graphs by making non-Gaussian assumptions about the data [24]. More precisely, it assumes that the error variables are non-Gaussian and independent, that each variable has a linear relationship, and that the causal graph is non-cyclic. There are two major types of LiNGAM: ICA-LiNGAM [24], which uses independent component analysis, and DirectLiNGAM [25], which uses regression analysis, but the former method tends to fall into local optimum and is dependent on parameters.

Several subsequent methods to improve the accuracy of LiNGAM have also been studied [10, 26]. An example is the approach of applying the kernel method to the independence measure of LiNGAM, which has the advantage of being able to estimate the causal structure with high accuracy even when the error variable assumed to be non-Gaussian is close to a Gaussian distribution or when the data have outliers [27]. As a further improvement, there is room to explore kernels that can construct independence measures with higher accuracy than the Gaussian kernel, a typical conventional kernel.

Since these above methods treat the search space of a causal graph as combinatorial, the number of candidate causal graphs increases super-exponentially as the increase of number of variables. Accordingly, as causal discovery algorithms that require less computational complexity, continuous optimization-based approaches have been developed in recent years. For example, non-combinatoric optimization via trace exponential augmented lagrangian structure learning is considered the first approach that recasts the combinatorial graph search problem into a continuous optimization problem with an acyclic constraint and uses gradient descent to determine the causal graph [28], and there have been several subsequent extensions of this approach [29–31]. For more information, see ref. [32]. On the other hand, compared to scale-invariant methods such as DirectLiNGAM, continuous optimization-based approaches are susceptible to rescaling of variables, such as standardization, and the risk of estimating different causal graphs depending on the scale of variables [33, 34] and the risk of detecting reversed edges between certain variables in causal graphs depending on the data have been pointed out [35]. Therefore, there could be room for improvement in the practical application of the constrained-based approach because it is difficult to know the true scale of the variables in advance, potentially limiting its application to real-world data.

Thus, while numerous types of causal discovery algorithms have been studied so far, tailored to specific situations and conducive to practice, causal discovery algorithms for small numbers of data have not been well researched, with a few exceptions [13, 36]. Also, to the best of my knowledge, there are no causal discovery algorithms that take advantage of quantum computing. Demonstrating the possibility of using quantum machine learning methods in causal discovery algorithms to estimate reasonable causal graphs with a small number of data could contribute to a broader range of medical applications of causal discovery algorithms.

### Aim

This research aims to develop a new causal discovery algorithm suitable for a small number of real-world medical data by utilizing quantum computing. More specifically, focusing on the commonality between quantum computing and kernel methods, which can both be viewed as efficient ways to perform computations in Hilbert space [19], a new algorithm called qLiNGAM is proposed, in which the quantum kernel is used for the independence measure of DirectLiNGAM [25]. To the best of my knowledge, this is the first study to apply quantum computing to LiNGAM. The performance of qLiNGAM in the low-data regime is evaluated using artificial data and further applied to real-world medical data to examine its performance and whether it can contribute to the detection of new medical knowledge. Furthermore, the feasibility of implementing qLiNGAM on real quantum hardware is discussed. This study shows the potential usefulness of utilizing quantum computing as a way to improve the performance of LiNGAM.

The rest of this article is structured as follows: The Methods section describes fundamental explanations of DirectLiNGAM, the kernel method, and the quantum kernel, followed by the presentation of qLiNGAM developed by combining them. The Results and Discussion section describes experiments in which qLiNGAM was applied to artificial data and real-world medical data, as well as experiments in which qLiNGAM was implemented on real quantum hardware, followed by discussions of each experimental result. The Conclusions section outlines the contributions and limitations of this study and directions for future work.

## Methods

### Causal discovery algorithm

On the basis of the findings of existing literature [27], DirectLiNGAM was more accurate for LiNGAM using a Gaussian kernel, and therefore, DirectLiNGAM was used as the basis for this study. In DirectLiNGAM with a total of *p* variables, a single regression analysis is performed *p*-1 times with each variable except a fixed *j*-th variable as an explanatory variable. Here, because it is known that an explanatory variable and a residual become independent when a single regression analysis in the correct direction is conducted [25], the explanatory variable at the time when the independence measure between the explanatory variable and the residual becomes the lowest is set as the parent node of the objective variable. Then, the same operation is performed on the other variables, except for the explanatory variable, and the arrows between the variables are sequentially determined. For more information on DirectLiNGAM, see refs. [25, 27].

### Independence measure

Improving the accuracy of DirectLiNGAM requires setting an appropriate independence measure. For the independence measure for DirectLiNGAM, the independence measure with a normalized cross-covariance operator (NOCCO) [37], which is independent of the shape of the kernel, was selected for a more appropriate application of the quantum kernel. More specifically, the fact that the Hilbert-Schmidt norm of the NOCCO approaches zero was used as a condition for a pair of random variables (*X*, *Y*) to be independent (i.e., *X*⫫*Y*).


$$I^{NOCCO}(X,Y)={∥V_{YX}∥}_{HS}^{2}$$ (1)

Here, *V*_*YX*_ is the NOCCO of *X* and *Y*, and ‖⋅‖_*HS*_ represents the Hilbert-Schmidt norm. If the variables *X* and *Y* are independent, *I*^*NOCCO*^(*X*, *Y*) in Eq (1) will be zero. Furthermore, given a finite sample (*X*_1_, *Y*_1_),……,(*X*_*n*_,*Y*_*n*_), the empirical independence measure ${\hat{I}}_{n}^{NOCCO}(X,Y)$ can be calculated as an estimator, as shown in Eqs (2) and (3) below.


$${\hat{I}}_{n}^{NOCCO}(X,Y)=Tr\left[R_{Y}R_{X}\right]$$ (2)

$$R_{Y}=G_{Y}{\left(G_{Y}+n\varepsilon_{n}I_{n}\right)}^{-1},R_{X}=G_{X}{\left(G_{X}+n\varepsilon_{n}I_{n}\right)}^{-1}$$ (3)

Here, *G*_*X*_ and *G*_*Y*_ are the centralized Gram matrices of each random variable and *ε*_*n*_ is the regularization constant. For a more detailed explanation of the independence measure with NOCCO, including the introduction of Eq (2), see ref. [37].

### Quantum computing and kernel method

**Fig 1:**
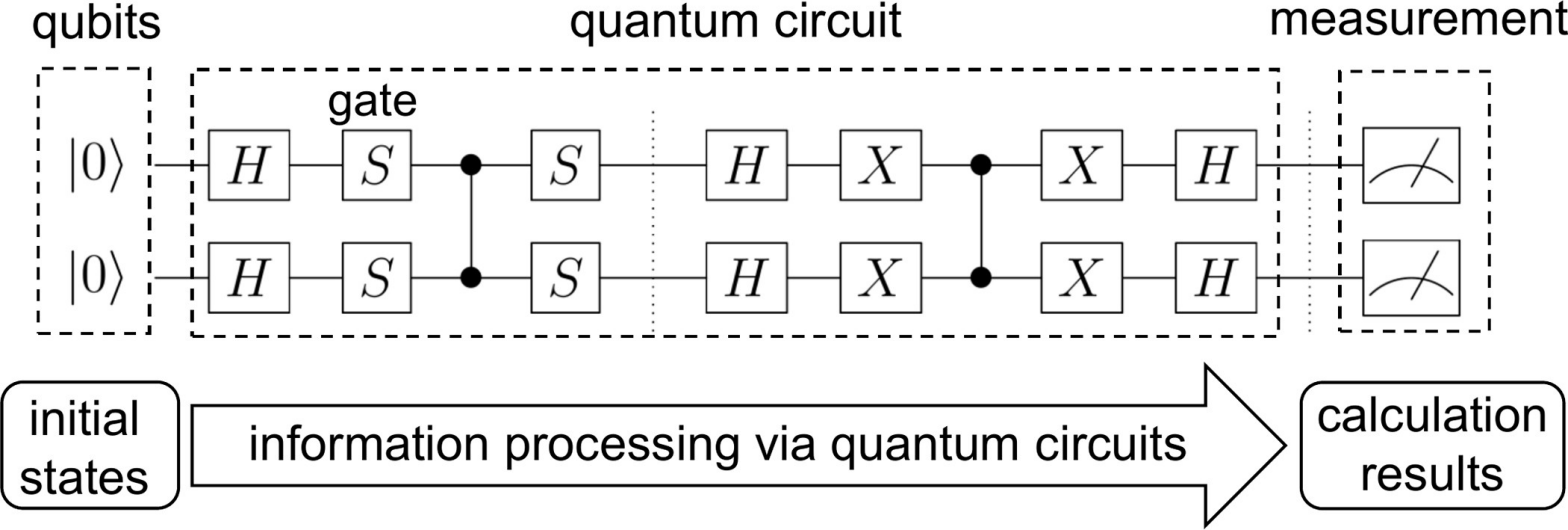
An example of quantum circuits. This shows a circuit that performs a quantum calculation called Grover’s algorithm. In quantum computation, qubits representing |0⟩^*n*^ as the initial states are prepared and passed through a quantum circuit to obtain the results. The wires mean that the quantum states are passed through as they are, and each gate marked with an alphabet changes the quantum state. The rightmost blocks with pictures of meters mean the measurement of the quantum state.

Recently, quantum computing has been attracting attention as an application technology for machine learning systems. Quantum computing uses qubits, which are superpositions of 0 and 1, and quantum circuits to process information. Fig 1 shows an example of a quantum circuit in a gate-based quantum computing framework, in which the quantum state represented by a qubit is passed through a quantum circuit to perform information processing.

Quantum kernel is a typical technique of machine learning algorithms for NISQ computing, which are hybrid algorithms for quantum computing and the kernel method. The kernel method uses a feature map Φ to transform a given data ***x***_*i*_ taken from the original space X to a higher-dimensional Hilbert space H.


$$\Phi :X\to H,x_{i}\to \Phi (x_{i})$$ (4)

The kernel method takes advantage of the fact that feature data mapping can be achieved without worrying about the dimensions of the feature space H. The basic idea of the kernel method is that it is not necessary to know the format of the feature map explicitly; instead, the overlap of points in the feature space is simply taken as a measure of similarity between the features of any two data samples, which is called the kernel function. More specifically, the similarity between data (*x*_*i*_, *x*_*j*_) in the feature space is expressed using the inner product ⟨⋅,⋅⟩ and the kernel function *k*(⋅,⋅) as shown in Eq (5) below.


$$k(x_{i},x_{j})=\langle \Phi (x_{i}),\Phi (x_{j})\rangle$$ (5)

The kernel function can also be expressed as a matrix in the form of a Gram matrix as follows:

$$K_{ij}=k(x_{i},x_{j})$$ (6)

Kernel methods embed data in a high-dimensional feature space to facilitate analysis, whereas quantum kernels use quantum circuits to construct the kernel. Quantum computers and kernel methods are very similar in principle in that they map information into a large space, but without the need for explicit computation in doing so. In the kernel method, access to the feature space is performed by the inner product of the kernels and feature vectors, whereas in the quantum computing, access to the Hilbert space of the quantum states is expressed by the inner product of quantum states. In the next section, the quantum kernels used in this study are discussed.

### Quantum kernel estimation

First, the kernel function of the feature mapping is related to the inner product of the quantum states and Eq (6) is expressed in terms of the quantum kernel as follows:

$$K_{ij}={\left|\left\langle \Phi (x_{j})|\Phi (x_{i})\right\rangle \right|}^{2}$$ (7)

where Φ(⋅) represents a quantum feature mapping and ⟨⋅|⋅⟩ represents the inner product of two quantum states in the Hilbert space. For the actual calculation of Eq (7) on a quantum computer, a quantum state Φ(***x***) needs to be created by acting a quantum gate *U*(***x***), which represents some unitary transformation, on the initial state |0⟩^*n*^ of the quantum circuit, and the quantum state is created as shown in the following equation:

$$U(x)|0\rangle^{n}=|\Phi (x)\rangle$$ (8)

One of the motivations for using quantum kernels in LiNGAM is to search for quantum kernels that are difficult to reproduce using a classical computer. To construct *U*(***x***) with a quantum circuit, the instantaneous quantum polynomial time (IQP) circuit was selected, which is known to have difficulty in accurately estimating the output probability distribution using a classical computer [38]. More specifically, under the assumption believed in computer science that the polynomial hierarchy does not collapse, it has been proven that the discrete probability distribution output from the IQP circuit cannot be sampled by classical computers even approximately in polynomial time [38]. Therefore, quantum kernels associated with the discrete probability distribution obtained by the IQP circuit are expected to have high expressive power that cannot be computed in polynomial time by classical computers [15].

**Fig 2:**
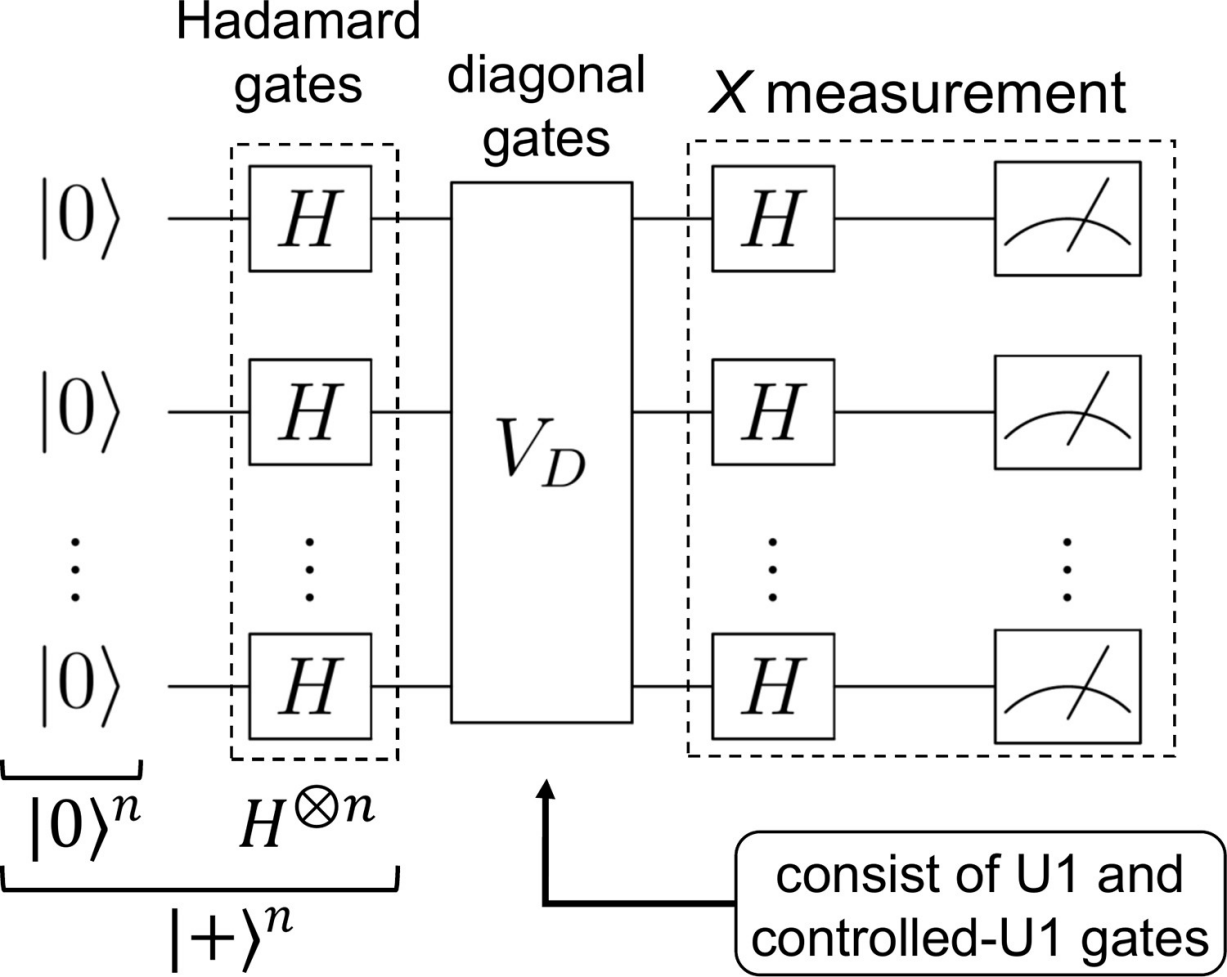
IQP circuit. First, the gates labeled *H* (Hadamard gate) are applied to the initial states of |0⟩^*n*^ to create the |+⟩^*n*^ state. Next, a gate (*V*_*D*_) consisting of diagonal matrices consisting of a high number of polynomials is applied. Finally, the *X* measurement is performed by applying again the gates labeled *H* and then performing the observation.

The IQP circuit is a circuit in which |0⟩^*n*^ as the initial states are multiplied by the Hadamard gates to create |+⟩^*n*^, which are then applied to the quantum gates consisting of only a few polynomial diagonal matrices, and finally, the *X* measurement is performed (Fig 2). If the IQP circuit is represented in mathematical terms, the layers that apply the Hadamard gates *H*^⊗*n*^ and that can be represented by a polynomial number of diagonal matrices *V*_*D*_(***x***) are repeated, as shown below.


$$U_{IQP}(x)=H^{⊗n}V_{D}(x)H^{⊗n}\cdots V_{1}(x)H^{⊗n}$$ (9)

In this study, the quantum gates *V*_*D*_(***x***), which consist of a polynomial number of diagonal matrices, were constructed in two layers: for one qubit, the *U*1 gate was adapted to all qubits, and then for two qubits, the controlled-*U*1 gate was linearly adapted to each neighboring qubit. The *U*1 and the controlled-*U*1 gates can be respectively expressed in matrix form as follows:

$$U1(\lambda )=\left(\begin{array}{cc} 1 & 0\\ 0 & e^{i\lambda } \end{array}\right)$$ (10)

$$controlled-U1(\lambda )=|0\rangle \langle 0|⊗I+|1\rangle \langle 1|⊗U1(\lambda )=\left(\begin{array}{cccc} 1 & 0 & 0 & 0\\ 0 & 1 & 0 & 0\\ 0 & 0 & 1 & 0\\ 0 & 0 & 0 & e^{i\lambda } \end{array}\right)$$ (11)

Here, the real data via the feature map function can be substituted for λ in Eqs (10) and (11) and implemented in the quantum circuit.

**Fig 3:**
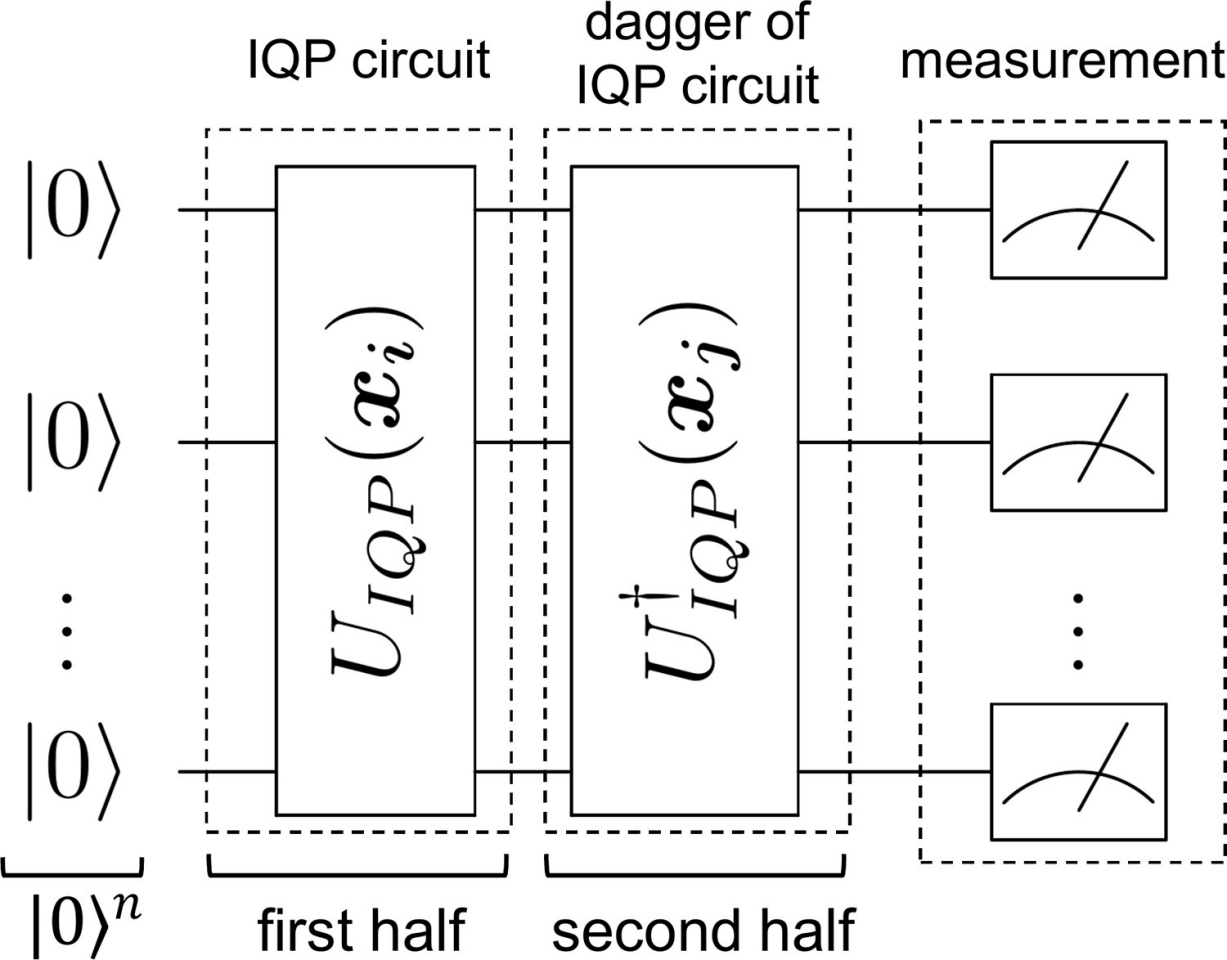
Inversion test. The circuit is divided into the first half and the second half, and the dagger of the first half is taken. *U*_*IQP*_(***x***) represents Eq (8), which consists of layers with Hadamard gates *H*^⊗*n*^ and a polynomial number of diagonal matrices *V*_*D*_(***x***).

For the quantum kernel estimation with a NISQ computer, there are two typical methods for quantum circuits: the swap test [39] and the inversion test [15]. In this study, the inversion test that required fewer gates was performed. Using the inversion test, each element of the Gram matrix is calculated through quantum circuits, and the proportion of the number of observations of 0^*n*^ out of the total number of measurements, respectively, is the element of the Gram matrix. Therefore, each element of the Gram matrix is always less than 1. Letting the total number of observations *R*, the value obtained by the inversion test gives an estimator for the Gram matrix up to a sampling error $\tilde{\epsilon }=O(R^{-1/2})$. To represent Eq (7) in a quantum circuit, in the inversion test, the circuit was divided into the first half and the second half and the dagger of the first half was taken and applied to the circuit in the second half (Fig 3). With the use of Eqs (7)-(9), the equation to be obtained can be expressed as follows:

$$K_{ij}={\left|\left\langle 0^{n}|U_{IQP}^{†}(x_{j})U_{IQP}(x_{i})|0^{n}\right\rangle \right|}^{2}$$ (12)

In summary, qLiNGAM is organized as follows: Eq (12) is used to calculate the Gram matrix in Eq (3) of the ${\hat{I}}_{n}^{NOCCO}$ calculation. The pseudocode for the algorithm is described in S1 Appendix.

## Results and discussion

### Preliminary experiment settings and model tuning

**Fig 4:**
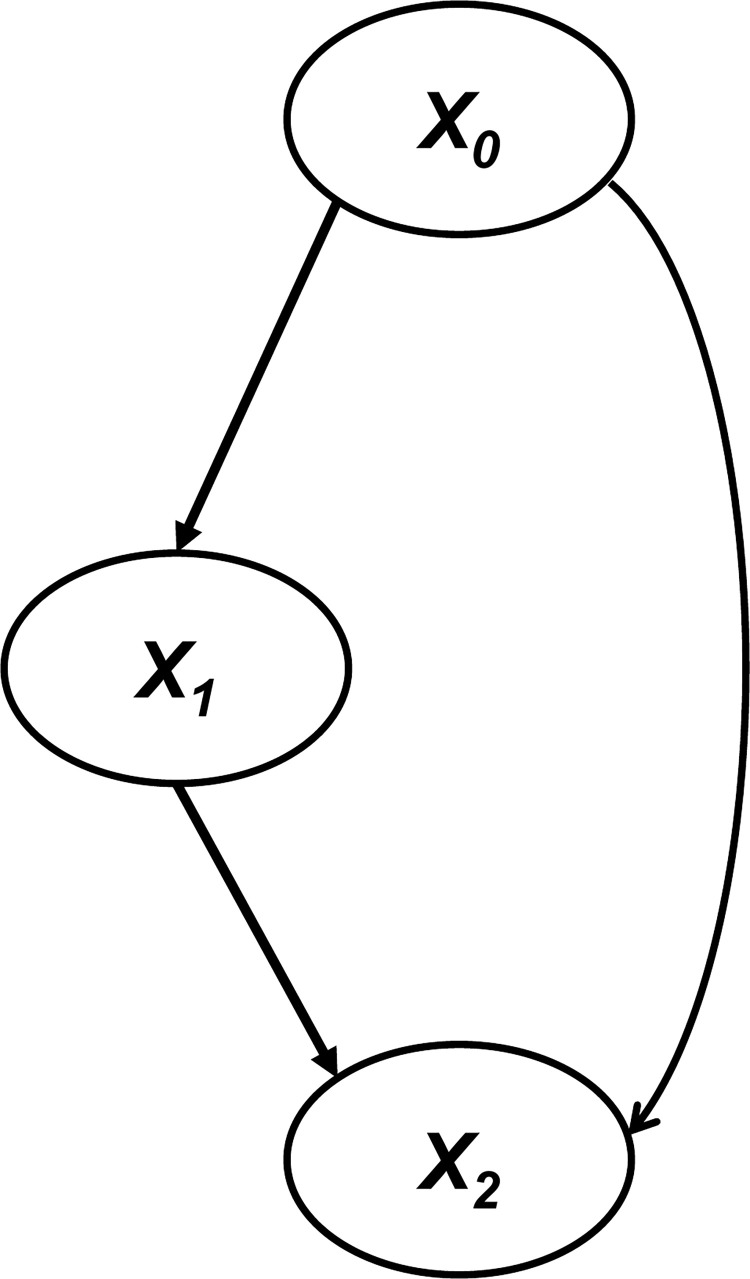
Causal relationship based on the artificial dataset generated from Eq (13). The ellipses represent the variables, and the arrows represent the directions of causality.

First, a simulation and model tuning using artificial data was conducted. With a sample size of 100 and 3 variables, a dataset with an error term *e* generated from the Laplace distribution (*μ* = 0, *λ* = 1) was created. The relationship between the three variables is as shown in Eq (13), which is represented in Fig 4. A total of 100 data sets with the structure of Eq (13) were prepared by changing the random seed and the error term.


$$\begin{array}{l} X_{0}=e\\ X_{1}=0.3\times X_{0}+e\\ X_{2}=0.3\times X_{1}+0.3\times X_{0}+e\\ e∼\frac{1}{2\lambda }exp(-\frac{\left|x-\mu \right|}{\lambda }) \end{array}$$ (13)

qLiNGAM was applied to 100 artificial datasets to determine the number of datasets that could identify the same causal structure as in Fig 4. qLiNGAM and DirectLiNGAM with Gaussian kernel [27], a typical conventional kernel, were applied to each dataset, and the number of correct causal structures identified was compared. Note that the value of *σ*, a parameter of the Gaussian kernel, was taken from the publicly available source code [40].

The quantum kernel was calculated using an IQP circuit was selected, which is known to have difficulty in accurately estimating the output probability distribution using a classical computer [38]. In constructing an IQP circuit, it is important to select a feature map function, and in this study, the activation functions that are widely used in the field of neural networks were selected. Although sigmoid, tanh, and rectified linear unit are well-known activation functions, the *tanh-shrink* function was selected, which is an activation function with continuity, as shown in Eq (14), for use as the phase of the quantum gates. In addition, when the data were inputted into the feature map function, they were normalized to mean 0 and variance 1, and then doubled for data scaling.


tanh−shrink(x)=x−tanh(x) (14)

Based on the IQP circuit, the number of qubits to be used in the circuit and the number of layers of diagonal matrices to be repeated (called depth) are important variables in the construction of the quantum kernel. In this study, the number of qubits and number of depths were set to 5 and 2, respectively, which presented the best results using 10 data samples created using a random seed that were different from the aforementioned artificial data. For the implementation of the quantum kernel used in qLiNGAM, a quantum circuit was built with the Python library cirq [41] and quantum calculations were performed using qulacs [42].

Both qLiNGAM and DirectLiNGAM with Gaussian kernel were able to identify the correct causal structure shown in Fig 4 for 39 out of 100 artificial datasets, whereas for 38 datasets, neither was able to identify it. In 14 datasets, only qLiNGAM was able to identify the correct causal structure, and in 9 datasets, only DirectLiNGAM with Gaussian kernel was able to identify it. Thus, qLiNGAM was able to estimate 14 datasets of the causal structures that DirectLiNGAM with Gaussian kernel was unable to detect.

### Evaluation of qLiNGAM accuracy under various conditions

Next, the circuit configuration and associated parameters (the number of qubits is 5, the number of depths is 2) were fixed and the accuracy of qLiNGAM was evaluated under various conditions. The common random graph, the Erdos-Reny graph [43], which is a random graph whose edges are independently added with equal probability, was selected, and the average degree was set to 2. Given a random acyclic graph *B*∈{0, 1}^*d*×*d*^ from the Erdos-Reny graph with the average degree equal to 2, the weights *W* were generated uniformly in {-2, -0.5}∪{0.5, 2} following the previous study [28]. Here, *d* denotes the number of variables, and in this experiment, was set to {5, 10, 15}. Given the weights *W*, random datasets *X* were sampled that satisfied the equation *X* = *W*^*T*^*X*+*e* according to the following two noise model: Exponential noise *e*~Exp(1); Gumbel noise *e*~Gumbel (0, 1).

To evaluate qLiNGAM accuracy in the low-data regime, sample sizes *n* was set to {100, 150, 200}. qLiNGAM and DirectLiNGAM with Gaussian kernel were applied to each dataset to compare their performance using the following four graph metrics: False discovery rate (FDR); True positive rate (TPR); False positive rate (FPR); Structural Hamming distance (SHD). For more information on the graph metrics, see ref. [28].

Table 1 shows the experimental results. When Exponential was the noise model, all the SHD values presented by qLiNGAM were lower than or equal to those of DirectLiNGAM with Gaussian kernel, except for *n* = 150, *d* = 15. All the TPR values presented by qLiNGAM were higher than or equal to those of DirectLiNGAM with Gaussian kernel. When Gumbel was the noise model, all the SHD values presented by qLiNGAM were lower than or equal to those of DirectLiNGAM with Gaussian kernel, except for *n* = 100, *d* = 5 and *n* = 200, *d* = 15. All the TPR values presented by qLiNGAM were higher than or equal to those of DirectLiNGAM with Gaussian kernel, except for *n* = 100, *d* = 5.

**Table 1:** Comparison of the accuracy of qLiNGAM and DirectLiNGAM with Gaussian kernel using the artificial data from the Erdos-Reny graph.

noise model	n	d	qLiNGAM				DirectLiNGAM with Gaussian kernel			
			FDR	TPR	FPR	SHD	FDR	TPR	FPR	SHD
Exponential	100	5	0.00	0.80	0.00	2	0.13	0.70	1.00	3
		10	0.47	0.45	0.32	17	0.65	0.35	0.52	21
		15	0.21	0.77	0.08	13	0.40	0.60	0.16	22
	150	5	0.00	0.80	0.00	2	0.00	0.80	0.00	2
		10	0.30	0.70	0.24	12	0.60	0.40	0.48	22
		15	0.59	0.53	0.31	30	0.58	0.47	0.25	29
	200	5	0.00	0.80	0.00	2	0.00	0.80	0.00	2
		10	0.22	0.70	0.16	10	0.61	0.35	0.44	22
		15	0.20	0.80	0.08	12	0.42	0.63	0.19	22
Gumbel	100	5	0.14	0.60	1.00	4	0.13	0.70	1.00	3
		10	0.29	0.60	0.20	12	0.63	0.35	0.48	20
		15	0.22	0.70	0.08	12	0.24	0.63	0.08	16
	150	5	0.00	0.80	0.00	2	0.00	0.80	0.00	2
		10	0.50	0.50	0.40	18	0.63	0.35	0.48	20
		15	0.23	0.80	0.09	13	0.42	0.60	0.17	22
	200	5	0.00	0.80	0.00	2	0.00	0.80	0.00	2
		10	0.25	0.60	0.16	11	0.63	0.35	0.48	20
		15	0.61	0.50	0.31	32	0.56	0.50	0.25	28

n, sample size; d, the number of variables; FDR, False discovery rate; TPR, True positive rate; FPR, False positive rate; SHD, Structural Hamming distance.

The results suggest that the use of quantum kernels may improve the performance of DirectLiNGAM. To further scrutinize this possibility statistically, exact Wilcoxon signed-rank tests were performed on four graph metrics, FDR, TPR, FPR, and SHD, to compare qLiNGAM and DirectLiNGAM with Gaussian kernel. The exact Wilcoxon signed-rank test results showed that qLiNGAM statistically significantly outperformed DirectLiNGAM with Gaussian kernel for all four graph metrics (p-values: FDR, 0.002; TPR, 0.001; FPR, 0.002; and SHD, 0.006). The improvement in not only the SHD but also the TPR suggests that applying the quantum kernel to DirectLiNGAM could lead to the identification of more correct arrows, which is favorable for the goal of finding causal relationships. Furthermore, the improvement in FPR suggests that wrong arrows are less likely to be identified using qLiNGAM, which seems suitable for the medical field, where mistakes are difficult to tolerate.

### Experiments with real-world medical data: Part 1

In addition, qLiNGAM was applied to real-world medical data. The circuit configuration and associated parameters were exactly the same as those of qLiNGAM designed for the artificial data, and cirq and qulacs were used for calculation. The first dataset used was the UCI Heart Disease Data Set [44], which is an open-source dataset in the field of cardiology. From the UCI Heart Disease Data Set, three continuous variables were used: ‘age,’ a variable representing age; ‘chol,’ a variable representing serum cholesterol in mg/dl, and ‘trestbps,’ a variable representing resting blood pressure in mmHg on admission to the hospital.

Since the p-values of the Shapiro-Wilk tests were less than 0.05 as well as confirmed by quantile-quantile plots, non-Gaussian assumptions would hold for all three variables. It was also assumed that ‘age’ would have approximately a linear effect on the other two variables. No unobserved confounding factors were assumed. Two datasets were prepared in advance: a full dataset of 297 cases from which records containing missing values were deleted, and a short version of the dataset from which 100 cases were randomly selected. For the descriptive statistics of these two data sets, the median, the first quartile, and the third quartile for each variable were as follows: for the full version of the data, ‘age,’ 56 [48–61], ‘chol,’ 243 [211–276], ‘trestbps,’ 130 [120–140]; for the short version of the data, ‘age,’ 55.5 [50.75–60], ‘chol,’ 236 [208.5–271.5], ‘trestbps,’ 130 [120–140].

**Fig 5:**
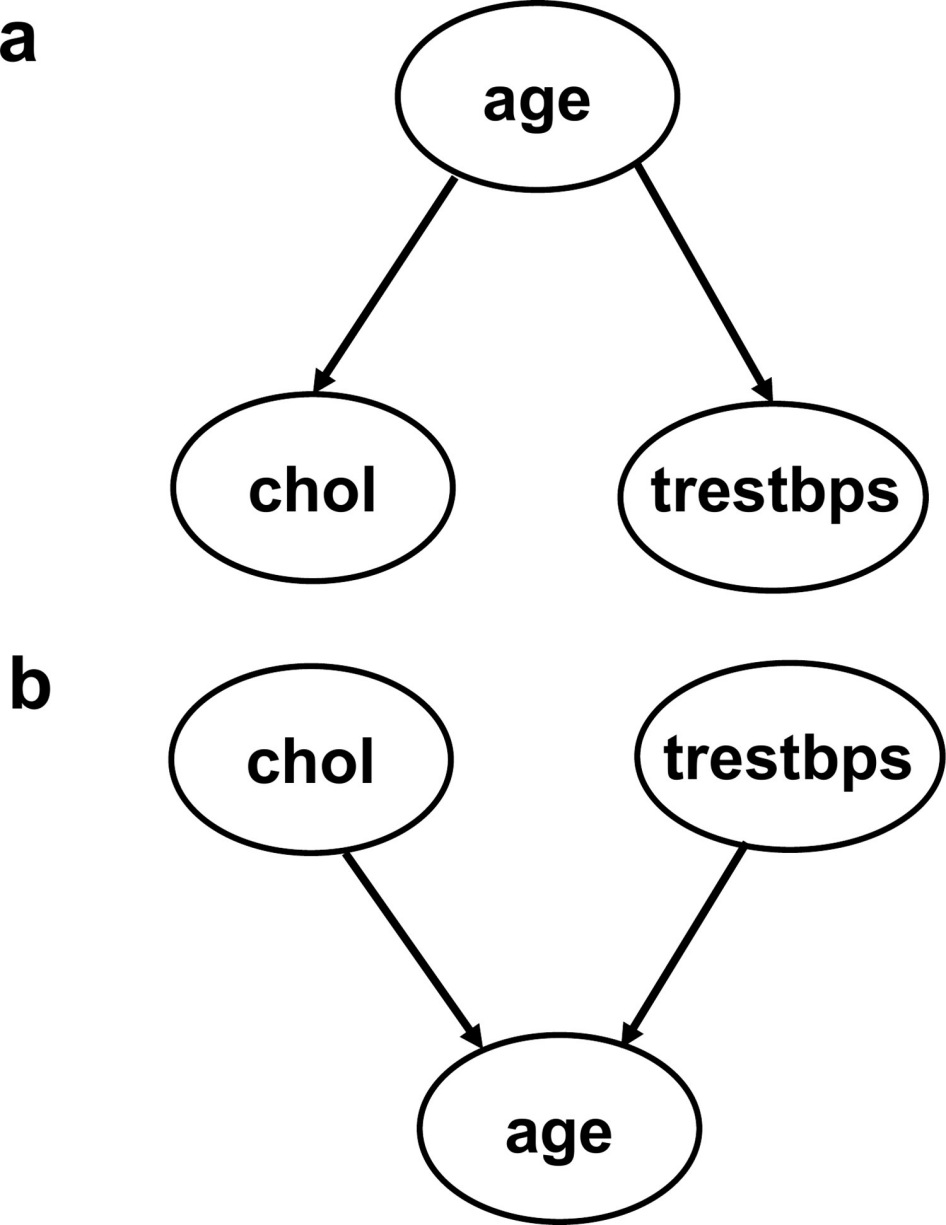
Experimental results using the UCI Heart Disease Data Set. The ellipses represent the variables, and the arrows represent the directions of causality. (a) A valid causal structure for UCI Heart Disease Data Set, because of arrows drawn from ‘age’ to ‘chol’ and ‘trestbps’. (b) Not a valid causal structure for UCI Heart Disease Data Set, because of arrows drawn from ‘chol’ and ‘trestbps’ to ‘age’. ‘age’, a variable representing age; ‘chol’, a variable representing serum cholesterol in mg/dl; ‘trestbps’, a variable representing resting blood pressure in mmHg on admission to the hospital.

The experiment results showed that qLiNGAM identified the causal relationship described in Fig 5A whether using the full version of the data with all 297 cases or the short version with only 100 cases. In this relationship, arrows are drawn from ‘age’ to ‘chol’ and ‘trestbps,’ which means that a valid relationship was detected. By contrast, DirectLiNGAM with Gaussian kernel identified the causal relationship described in Fig 5B whether using the full version of the data with all 297 cases or the short version with only 100 cases. The relationship in Fig 5B is not a valid result, where arrows are drawn from ‘chol’ and ‘trestbps’ to ‘age’. Given that the data characteristics of the full version of the data and the short version of the data are close, these experimental results show the case where qLiNGAM was able to identify valid causal relationships that cannot be identified by DirectLiNGAM with Gaussian kernel, even with a smaller amount of data.

#### Discussion on the contribution of using quantum kernels

Although it is not fully clear why qLiNGAM was able to estimate the causal structure that cannot be estimated by existing algorithms using Gaussian kernels, it is possible that the Gram matrices generated using quantum calculation with an IQP circuit, which have significantly different values than those of the Gaussian kernels, allowed the evaluation of the independence with higher accuracy. With respect to this issue, *g*_*CQ*_ is a known geometric difference for quantitatively evaluating the difference between quantum kernels and conventional kernels, as shown in Eq (15) below [45].

$$g_{CQ}=g(K^{C}∥K^{Q})=\sqrt{{∥\sqrt{K^{Q}}{\left(K^{C}\right)}^{-1}\sqrt{K^{Q}}∥}_{\infty }}$$ (15)

Here, *K*^*C*^ and *K*^*Q*^ represent the Gram matrix of the conventional kernels and the quantum kernels, respectively, and ‖⋅‖_∞_ represents the spectral norm. A large value of *g*_*CQ*_ means that the difference between the two kernels is large.

For the full version of the data with all 297 cases, *g*_*CQ*_ was used to evaluate the relationships from ‘age’ to ‘chol’ and ‘trestbps,’ which were identified by qLiNGAM but not by DirectLiNGAM with Gaussian kernel. More specifically, a single regression analysis was performed among each variable, and *g*_*CQ*_ was calculated for the explanatory variable and the residual, respectively. Compared to a single regression analysis in the correct direction, such as from ‘age’ to ‘chol’ or from ‘age’ to ‘trestbps,’ when a single regression analysis was performed in the opposite direction, *g*_*CQ*_ of the residual differed remarkably, while *g*_*CQ*_ of the explanatory variable differed little. More detailed results are provided in S1 Table.

One possible explanation is that the differences between the Gram matrices of quantum kernels and Gaussian kernels for the residuals, in particular, may have affected the differences in the independence measure. While *g*_*CQ*_ has been proposed as a metric to assess the quantum advantage of quantum kernels regarding prediction performance [45], *g*_*CQ*_ might also be available to assess whether quantum kernels are useful for independence measures. However, the numerical results of the obtained *g*_*CQ*_ in S1 Table might have been unstable, especially concerning the calculation of the inverse matrix of *K*^*C*^ in Eq (15), due to the very high condition number of the matrix (10^21^−10^31^), thus the correctness of this interpretation needs to be verified. In the future, a more elaborate analysis of the properties of independence measures using quantum kernels is needed, such as a quantitative evaluation of these properties through a permutation test.

### Experiments with real-world medical data: Part 2

The next real-world medical data source applied to qLiNGAM was the Pima Indians Diabetes Database [46], an open-source dataset for diabetic diseases. The circuit configuration and associated parameters were exactly the same as those of the first experiment. From the Pima Indians Diabetes Database, three continuous variables were used: ‘age,’ a variable for age; ‘insulin,’ a variable for insulin concentration 2 h after the oral glucose tolerance test; and ‘glucose,’ a variable for blood glucose concentration 2 h after the oral glucose tolerance test.

Since the p-values of the Shapiro-Wilk tests were less than 0.05 as well as confirmed by quantile-quantile plots, non-Gaussian assumptions would hold for all three variables. It was also assumed that ‘age’ would have approximately a linear effect on the other two variables. For the relationship between ‘insulin’ and ‘glucose,’ an approximately linear relationship was assumed, but it was not easy to clinically determine which affects which. No unobserved confounding factors were assumed. Two datasets were prepared in advance: a full dataset of 392 cases from which records containing missing values were deleted, and a short version of the dataset from which 100 cases were randomly selected. For the descriptive statistics of these two data sets, the median, the first quartile, and the third quartile for each variable were as follows: for the full version of the data, ‘age,’ 27 [23–36], ‘insulin,’ 125 [76.75–190], ‘glucose,’ 119 [99–143]; for the short version of the data, ‘age,’ 26.5 [23–37], ‘insulin,’ 120 [84.25–180], ‘glucose,’ 115 [96.75–142.25].

**Fig 6:**
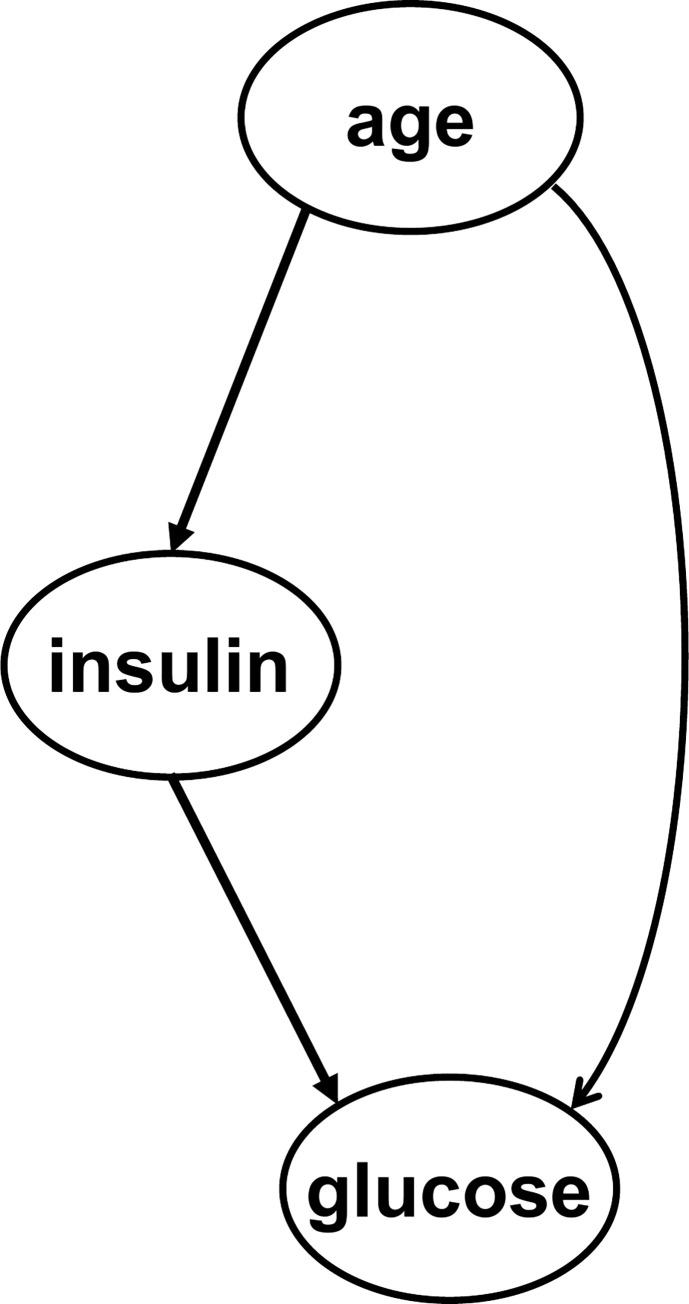
Experimental results using the Pima Indians diabetes database. The ellipses represent the variables, and the arrows represent the directions of causality. ‘insulin’, a variable for insulin concentration 2 h after the oral glucose tolerance test; and ‘glucose’, a variable for blood glucose concentration 2 h after the oral glucose tolerance test.

The results of the experiment showed that both qLiNGAM and DirectLiNGAM with Gaussian kernel identified the causal relationship in Fig 6. for both the full example dataset (392 examples) and the short version dataset (100 randomly selected examples). As shown in Fig 6, the left-hand path shows that ‘age’ affects ‘insulin,’ which, in turn, affects ‘glucose,’ and the right-hand path shows that ‘age’ directly affects ‘glucose’. Because it was assumed that there were other factors between age and insulin concentration that were not used in this study, such as obesity, the clinically valid relationship can be confirmed, which is depicted by the right-hand path, where age affects blood glucose via a path other than insulin concentration. Thus, a case where qLiNGAM can present a causal structure even for cases that are not easy to determine clinically at first glance has been confirmed. While qLiNGAM was not the only method that could estimate valid causal relationships in this case, the results suggest that qLiNGAM may be a good choice among causal discovery algorithms, including existing algorithms for a small number of medical data.

### Feasibility of implementing qLiNGAM on real quantum hardware

Finally, the possibility of implementation of qLiNGAM on real quantum hardware is discussed. The real quantum hardware selected in this study was the ibm_kawasaki 27-qubit quantum device. Qiskit [47], a python library, was used to access this device.

While it would be preferable to set the circuit configuration and associated parameters exactly the same as those in previous experiments, it is difficult to obtain correct results when using existing real quantum hardware. More specifically, when the number of qubits is 5 and the number of depths is 2, it is difficult to obtain correct calculation results due to the large influence of errors. Although ibm_kawasaki is real quantum hardware with relatively small errors compared to other devices in IBM Quantum, it was assumed that setting the number of qubits to 4 and the number of depths to 1 would be reasonable for the implementation of quantum kernels, considering the current errors. Among the 27 qubits in ibm_kawasaki, four linearly connected qubits (specifically, q0, q1, q4, and q7) were selected to try to reduce the influence of various errors that occur in real quantum hardware on calculation accuracy. In addition, to mitigate the readout error of each qubit, the readout error mitigation routine in Qiskit was used to calculate the Gram matrices. In more detail, a 16×16 calibration matrix was prepared and applied to the obtained calculation results to correct the four qubit readout results. The number of measurement shots was set to 8192.

**Fig 7:**
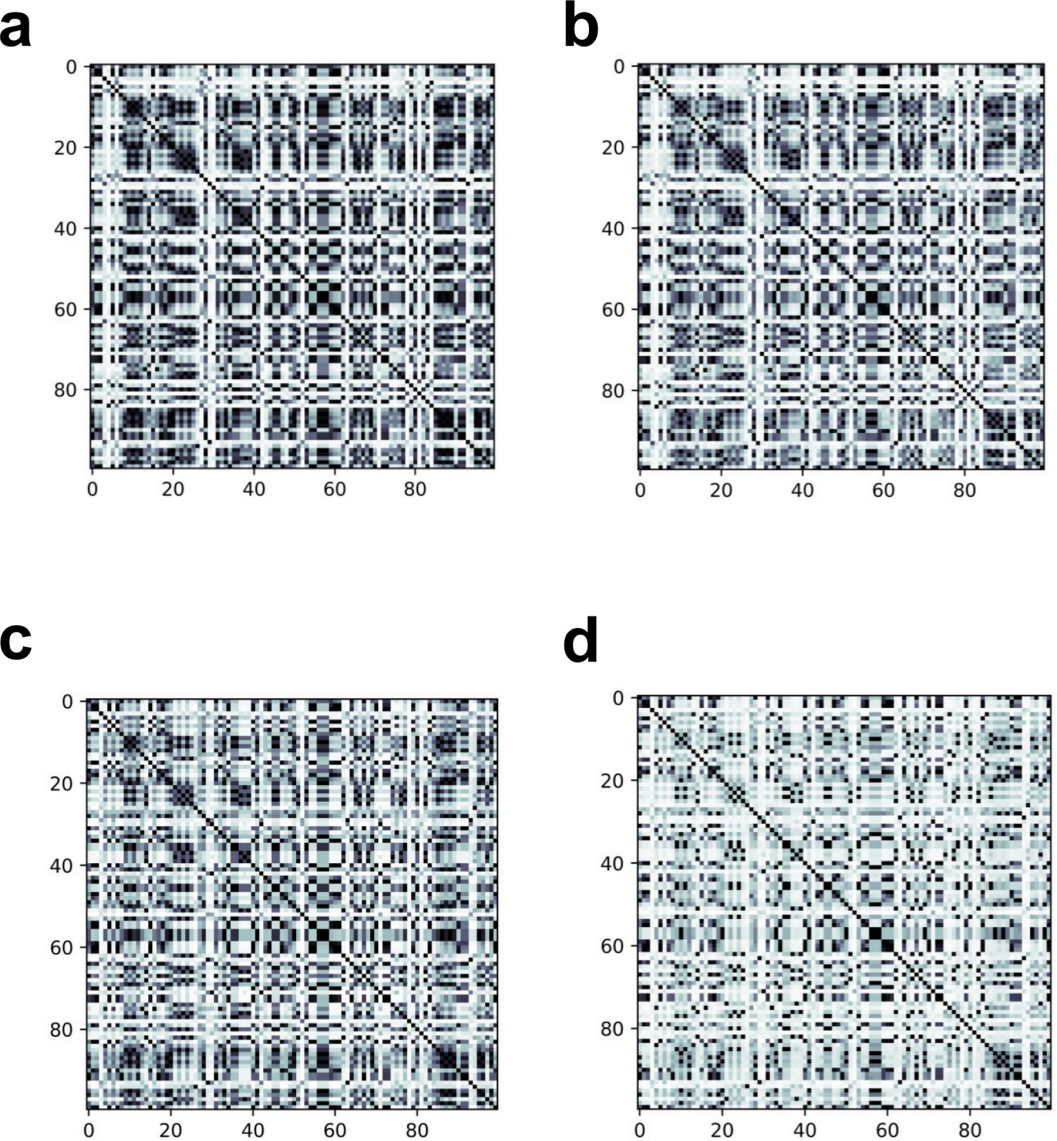
Gram matrices for 100 cases in the Pima Indians diabetes database using the variable ‘age’. Each element of the Gram matrix is normalized from 0 to 1. The stronger the black color, the closer it is to 1. (a) The Gram matrix of the quantum kernel with the number of qubits of 4 and the number of depths of 1. (b) The Gram matrix of the quantum kernel created using ibm_kawasaki with the number of qubits of 4 and the number of depths of 1. (c) The Gram matrix of the quantum kernel with the number of qubits of 5 and the number of depths of 2. (d) The Gram matrix of the quantum kernel created using ibm_kawasaki with the number of qubits of 5 and the number of depths of 2.

Fig 7 shows the Gram matrices using the variable ‘age’ in a randomly selected dataset of 100 cases from the Pima Indians Diabetes Database, which is the same as the short version of the dataset used in the experiment described above. It can be seen that the Gram matrix of the quantum kernel using the ibm_kawasaki is close to the theoretical values, due to the adjustment of the number of depths and qubits and the error mitigation methods (Fig 7A and 7B). However, when the Gram matrix of the quantum kernel was created using ibm_kawasaki with the number of qubits as 5 (specifically, q0, q1, q4, q6, and q7) and the number of depths as 2, it is different from the theoretical values due to the large influence of errors (Fig 7C and 7D).

Thus, qLiNGAM could be implemented on real quantum hardware depending on error conditions of the hardware in cases where the same causal structure can be estimated for the number of qubits of 4 and the number of depths of 1 as for the number of qubits of 5 and the number of depths of 2, such as when using the short version of the Pima Indians Diabetes Database. However, qLiNGAM would be difficult for the implementation on real quantum hardware in cases where setting the number of qubits to 4 and the number of depths to 1 results in an incorrect causal structure estimated, unlike when the number of qubits is 5 and the number of depths is 2, such as when using the short version of the UCI heart disease dataset.

In addition, S2 Table shows the results of the experiment performed in the previous section using the artificial data created from the Erdos-Reny graph, with the number of qubits changed to 4 and the number of depths changed to 1. From S2 Table, it can be seen that the SHD values were higher in most cases by setting the number of qubits to 4 and the number of depths to 1, especially when Gumbel was used as the noise model. Therefore, improvement of various errors of the devices is essential to implement qLiNGAM on real quantum hardware while maintaining accuracy, and future development of them is expected.

### Limitations and future perspectives

This study has several limitations that need to be solved in the future. The challenges related to quantum computing are listed below. First, although a quantum kernel based on the IQP circuit was constructed in this study, for this quantum kernel to gain the quantum advantage truly, it requires such a large number of qubits that it is difficult for conventional computers to simulate. However, it is known that, as the number of qubits increases, the Gram matrix of the quantum kernel becomes sparser and approaches the identity matrix, and improvement methods are being sought [45]. The main focus of this study is the proposal and demonstration of qLiNGAM, and further improvements are needed to introduce new knowledge of quantum kernels in the future. Second, the quantum circuit needs to be improved to design the quantum kernel. For example, it is not easy to decide whether the IQP circuit is really suitable and how many depths and qubits are appropriate. In addition, there is room for improvement in the selection of the feature map function and in the scaling of variables. In this study, the selection of such function was done heuristically; however, a theoretical background for the axis of search is desired in the future. Third, implementation on real quantum hardware is also an important issue. It is expected that the number of qubits and the error rate will be improved in the future, and it will be necessary to adjust qLiNGAM according to the improvement of the performance of real quantum hardware.

Several limitations in this study other than those mentioned above are listed below. First, since qLiNGAM is based on DirectLiNGAM, nonlinear relationships among variables and the existence of unobserved confounding factors are not considered in this study. In the future, to make the method in this study more applicable to a wider variety of real-world medical data, it will be necessary to apply quantum kernels to independence measures in, for example, additive noise model [48] for nonlinear relationships among variables or parceLiNGAM [49] that are robust to the effects of unobserved confounding factors. Second, this study focused only on the independence measure and did not examine the effect of the search algorithms. In a previous study [27], beam search was applied to DirectLiNGAM to improve its accuracy, and selecting a more appropriate search algorithm may improve the accuracy of qLiNGAM. In the future, it will be necessary to closely examine how each meta-heuristic method, ranging from traditional methods such as tabu search to newly developed methods [50–52], can be used for qLiNGAM. Third, because this study used a limited variety of real-world medical data sets, statistical evaluations of the accuracy of qLiNGAM on real-world medical data were not performed. In the future, the accuracy of qLiNGAM should be evaluated on a wider range of real-world medical data.

Much of the discovery of new medical knowledge has so far been generated empirically, and the experience of clinicians should continue to be respected in the future. By contrast, the amount of medical knowledge required for clinical practice is enormous, and it is becoming increasingly difficult to discover all new knowledge from clinicians’ experience alone. It is hoped that the results of this study will support clinicians’ hypothesis formation by detecting novel medical knowledge from real-world medical data. More specific applications are expected to include drug repositioning, such as the search for novel drug responses, causal search using an integrated database of genomic and clinical data, and detection of the relationship between lifestyle habits and disease outcomes in the health technology field. In particular, as sample collection can be very time-consuming and costly, applications of quantum machine learning in biology and medicine are expected to reduce the number of samples [53]. In the future, it is desirable to develop algorithms that can be used when the number of samples obtained is smaller than the number of variables obtained, such as real-world medical data on rare diseases.

## Conclusions

In this study, qLiNGAM was developed and applied to real-world medical data to verify whether valid causal structures could be identified. Furthermore, the possibility of implementation of qLiNGAM on real quantum hardware was discussed. When qLiNGAM was applied to real-world medical data, a case was confirmed in which the causal structure could be correctly estimated even when the amount of data was small, which was not possible with DirectLiNGAM with Gaussian kernels. It was suggested that qLiNGAM could be a good choice among causal discovery algorithms in the low-data regime for novel medical knowledge discovery.

The limitation of this study is that no experiments using quantum kernels to obtain a truly quantum advantage have been conducted. In the future, it is necessary to improve the way to apply quantum computing to DirectLiNGAM, including further development of quantum kernels and the design of quantum circuits that consider their implementation in quantum hardware. In addition, since qLiNGAM is based on DirectLiNGAM, situations assumed in real-world medical data, such as nonlinear relationships among variables and unobserved confounding factors, are not considered in this study. In the future, each method that relaxes the assumptions of LiNGAM and search algorithms such as meta-heuristics should be applied to qLiNGAM, and its accuracy should be examined on various real-world medical data.

## Supporting information

S1 Appendix The pseudocode for qLiNGAM. (PDF)

S1 Table Comparison of *g*_*CQ*_ for the explanatory variable and the residual. *g*_*CQ*_ is the geometric difference for quantitatively evaluating the difference between quantum kernels and conventional kernels. The explanatory variable is the tail in the directed edge. The residual means that of a single regression analysis of the head in the directed edge on the tail in the directed edge.
(PDF)

S2 Table Comparison of the accuracy of qLiNGAM with the number of qubits of 5 and the number of depths of 2 and qLiNGAM with the number of qubits of 4 and the number of depths of 1 using the artificial data from the Erdos-Reny graph. (PDF)
