# Supplementary material for: Application of quantum computing to a linear non-Gaussian acyclic model for novel medical knowledge discovery
Source: PLoS One. 2023 Apr 5;18(4):e0283933. doi: 10.1371/journal.pone.0283933 (PMC10075477; doi:10.1371/journal.pone.0283933)
Supplement: S1 Appendix — (PDF) [file pone.0283933.s001.pdf]

S1 Appendix

---

qLiNGAM

---

1) Input:

   $p \times n$ data matrix $\boldsymbol{X}$,

   set $\boldsymbol{U}$ of the subscripts of all $x_i \in \boldsymbol{X}$,

   initialized ordering list of variables $\boldsymbol{K} = \phi$ and $m := 1$.

2) Repeat until $p - 1$ subscripts are added to $\boldsymbol{K}$.

   a) Regress x_i on x_j for all $i \in \boldsymbol{U} - \boldsymbol{K} (i \neq j)$ and derive the residual data matrix $\boldsymbol{R}_j$ from the data matrix $\boldsymbol{X}$ for all $j \in \boldsymbol{U} - \boldsymbol{K}$.

   b) Find a top node variable x_t using the independence measure in equations (2) and (3):

$$x_t = arg \min_{j \in U - K} T(x_j; \boldsymbol{U} - \boldsymbol{K}),$$

$$T\left(x_j; \boldsymbol{U}\right) = {}_{i \in U, i \neq j} \, I_n^{NOCCO}(x_j, r_i^{(j)}),$$

   where the Gram matrices in $I_n^{NOCCO}(x_j, r_i^{(j)})$ are calculated using the quantum circuits in equation (12).

   c) Add the subscript t of the variable x_t to $\boldsymbol{K}$.

   d) Let $\boldsymbol{X} := \boldsymbol{R}_{(t)}$ and $m := m + 1$.

3) Add the subscript of the remaining variable to $\boldsymbol{K}$.

4) Construct the strictly lower triangular structure of the connection matrix $\boldsymbol{B}$ using $\boldsymbol{K}$.

5) Estimate the connection strength b_{ij}, which is an element of $\boldsymbol{B}$ using the data matrix $\boldsymbol{X}$.
