# Supplementary material for: Application of quantum computing to a linear non-Gaussian acyclic model for novel medical knowledge discovery
Source: PLoS One. 2023 Apr 5;18(4):e0283933. doi: 10.1371/journal.pone.0283933 (PMC10075477; doi:10.1371/journal.pone.0283933)
Supplement: S1 Table — gCQ is the geometric difference for quantitatively evaluating the difference between quantum kernels and conventional kernels. The explanatory variable is the tail in the directed edge. The residual means that of a single regression analysis of the head in the directed edge on the tail in the directed edge. (PDF) [file pone.0283933.s002.pdf]

S1 Table

directed edge	g_{CQ} for the explanatory variable	g_{CQ} for the residual
‘age’ → ‘chol’	5.76	5.94
‘chol’ → ‘age’	4.82	2753042.03
‘age’ → ‘trestbps’	5.76	6649.10
‘trestbps’ → ‘age’	3.36	2042716.36

‘age’, a variable representing age; ‘chol’, a variable representing serum cholesterol in mg/dl; ‘trestbps’, a variable representing resting blood pressure in mmHg on admission to the hospital
