# Supplementary material for: Application of quantum computing to a linear non-Gaussian acyclic model for novel medical knowledge discovery
Source: PLoS One. 2023 Apr 5;18(4):e0283933. doi: 10.1371/journal.pone.0283933 (PMC10075477; doi:10.1371/journal.pone.0283933)
Supplement: S2 Table — (PDF) [file pone.0283933.s003.pdf]

S2 Table

noise model	n	d	qLiNGAM qubit=5, depth=2				qLiNGAM qubit=4, depth=1			
			FDR	TPR	FPR	SHD	FDR	TPR	FPR	SHD
Exponential	100	5	0.00	0.80	0.00	2	0.00	0.80	0.00	2
		10	0.47	0.45	0.32	17	0.45	0.55	0.36	15
		15	0.21	0.77	0.08	13	0.42	0.60	0.17	21
	150	5	0.00	0.80	0.00	2	0.29	0.50	2.00	5
		10	0.30	0.70	0.24	12	0.30	0.70	0.24	12
		15	0.59	0.53	0.31	30	0.61	0.43	0.27	30
	200	5	0.00	0.80	0.00	2	0.00	0.80	0.00	2
		10	0.22	0.70	0.16	10	0.91	0.10	0.80	29
		15	0.20	0.80	0.08	12	0.56	0.53	0.27	29
Gumbel	100	5	0.14	0.60	1.00	4	0.14	0.60	1.00	4
		10	0.29	0.60	0.20	12	0.78	0.20	0.56	27
		15	0.22	0.70	0.08	12	0.73	0.33	0.36	36
	150	5	0.00	0.80	0.00	2	0.57	0.30	4.00	7
		10	0.50	0.50	0.40	18	0.78	0.20	0.56	25
		15	0.23	0.80	0.09	13	0.62	0.50	0.32	34
	200	5	0.00	0.80	0.00	2	0.14	0.60	1.00	4
		10	0.25	0.60	0.16	11	0.67	0.35	0.56	22
		15	0.61	0.50	0.31	32	0.56	0.47	0.24	28

n, sample size; d, the number of variables; FDR, False discovery rate; TPR, True positive rate; FPR, False positive rate; SHD, Structural Hamming distance.
